# Real-world effectiveness and safety of imeglimin: a single-center retrospective cohort study in Japan

**DOI:** 10.3389/fcdhc.2025.1694522

**Published:** 2025-12-16

**Authors:** Taro Fujisawa, Takehiro Kato, Kazuhisa Takami, Shinya Fukuda, Risako Imai, Tomoya Kawashima, Ryosuke Horita, Katsuhisa Sakai, Akiko Yamada, Shin Tsunekawa, Daisuke Yabe

**Affiliations:** 1 Department of Diabetes, Endocrinology and Metabolism, Gifu University Graduate School of Medicine, Gifu, Japan; 2 Department of Rheumatology and Clinical Immunology, Gifu University Graduate School of Medicine, Gifu, Japan; 3 Department of Endocrinology and Metabolism/Diabetes Center, Central Japan International Medical Center, Minokamo, Japan; 4 Center for One Medicine Innovative Translational Research, Gifu University Institute for Advanced Studies, Gifu, Japan; 5 Department of Diabetes, Endocrinology and Nutrition, Kyoto University Graduate School of Medicine, Kyoto, Japan

**Keywords:** type 2 diabetes, imeglimin, real-world evidence, older adults, effectiveness and safety

## Abstract

**Aims/Introduction:**

This study aimed to evaluate the real-world effectiveness and safety of imeglimin in individuals with type 2 diabetes.

**Materials and methods:**

We retrospectively reviewed 79 individuals (52 men, 27 women) with type 2 diabetes who newly initiated imeglimin (1,000 mg twice daily) and were followed for 12 months at the Central Japan International Medical Center between September 2022 and December 2023. Individuals were stratified by age (<65, 65–74, ≥75 years) and by the presence or absence of biguanide dose reduction at imeglimin initiation. The primary endpoint was the change in HbA1c from baseline to 12 months. Secondary endpoints included the achievement rate of glycemic targets, incidence of adverse events, and changes in body weight, blood pressure, liver and renal function, lipid profile, and uric acid.

**Results:**

HbA1c significantly decreased one month after initiation and the improvement was sustained through 12 months (mean change from baseline -0.8 ± 1.2%). Effectiveness and safety did not differ significantly among age groups. Gastrointestinal symptoms were the most common adverse events (21.5%), with no age-related differences. HbA1c reduction was greater in individuals without biguanide dose reduction compared with those with dose reduction (–1.5 ± 1.7% *vs* –0.5 ± 0.7%, p=0.019), although adverse event frequency was comparable. Importantly, gastrointestinal disturbances were more frequent when imeglimin was combined with metformin ≥1,000 mg/day (p=0.032). Significant reductions were also observed in body weight, triglycerides, and liver enzymes at 12 months.

**Conclusions:**

Imeglimin demonstrated sustained glycemic effectiveness and favorable tolerability in real-world practice, including among elderly individuals with type 2 diabetes. These findings suggest imeglimin as a valuable therapeutic option for older adults with type 2 diabetes. Caution is warranted when co-administered with high-dose metformin, whereas combination with <1,000 mg/day appears relatively safe.

## Introduction

Imeglimin is a novel oral glucose-lowering agent with a tetrahydrotriazine structure, first approved in Japan in September 2021. Its glucose-lowering efficacy is mediated through dual mechanisms: 1) enhancement of glucose-dependent insulin secretion, and 2) improvement of hepatic glucose metabolism via mitochondrial modulation (1, 2). Since it has been well recognized that type 2 diabetes in east Asians is primarily characterized by impaired insulin secretion (3), phase 3 clinical trials, the Trials of Imeglimin for Efficacy and Safety (TIMES 1–3), demonstrated both efficacy and safety of imeglimin in Japanese individuals with type 2 diabetes (4–6). Nevertheless, evidence from real-world clinical practice remains scarce, particularly among older adults with type 2 diabetes, a population that continues to expand and is estimated to account for 29% of the global diabetes population (7). Of particular importance is the real-world safety profile of imeglimin when co-administered with biguanides. Biguanides are inexpensive and supported by strong evidence for preventing the development and progression of diabetes-related complications. However, the TIMES 2 trial reported a higher incidence of gastrointestinal disturbances with the combination of imeglimin and biguanides (5), underscoring the need to establish safe dosing strategies. Against this background, the present retrospective study aimed to assess the real-world effectiveness and safety of imeglimin, stratified by age group and by the presence or absence of biguanide dose reduction at treatment initiation.

## Materials and methods

### Study design

This retrospective observational study included individuals aged ≥16 years with type 2 diabetes who newly initiated imeglimin (1,000 mg twice daily; total daily dose 2,000 mg) at the Central Japan International Medical Center between September 1, 2022, and December 31, 2023. Clinical data from routine practice were collected over a 12-month follow-up period after treatment initiation. The study protocol was approved by the Ethics Committee of the Central Japan International Medical Center (Approval No. 2023-027). Informed consent was obtained through an opt-out procedure on the hospital website, in accordance with the Declaration of Helsinki.

### Stratification and endpoints

Participants were stratified into three age groups (<65, 65–74, and ≥75 years) and by the presence or absence of biguanide dose reduction at imeglimin initiation. The primary endpoint was the change in HbA1c from baseline to 12 months. Secondary endpoints included the proportion of patients achieving glycemic targets (defined as HbA1c <7.0% for individuals <65 years, and for those ≥65 years, according to the joint recommendations of the Japanese Diabetes Society and the Japan Geriatrics Society (8, 9)), the incidence of adverse events, and changes in body weight, blood pressure, heart rate, liver function, renal function, lipid profile, and serum uric acid.

### Statistical analysis

Statistical analyses were performed using Easy R (EZR version 1.61). Comparisons of baseline characteristics across age groups and between individuals with and without biguanide dose reduction were conducted using unpaired t-tests, the Kruskal–Wallis test, and Fisher’s exact test. Within-group changes in body weight, blood pressure, heart rate, and laboratory parameters before and after imeglimin initiation were assessed using the Wilcoxon signed-rank test, and between-group comparisons were evaluated with the Mann–Whitney U test. Differences in glycemic target achievement rates by age group and biguanide dose reduction, as well as associations between biguanide dose reduction and gastrointestinal adverse events and between metformin dose and gastrointestinal adverse events, were examined using Fisher’s exact test. Changes in HbA1c were assessed by repeated-measures ANOVA, stratified by age group and by biguanide dose reduction. *Post hoc* pairwise comparisons were not conducted, as the analysis aimed to evaluate the overall time effect rather than differences between individual time points. All values are expressed as mean ± SD. A P-value <0.05 was considered statistically significant.

## Results

### Study population

**Figure 1:**
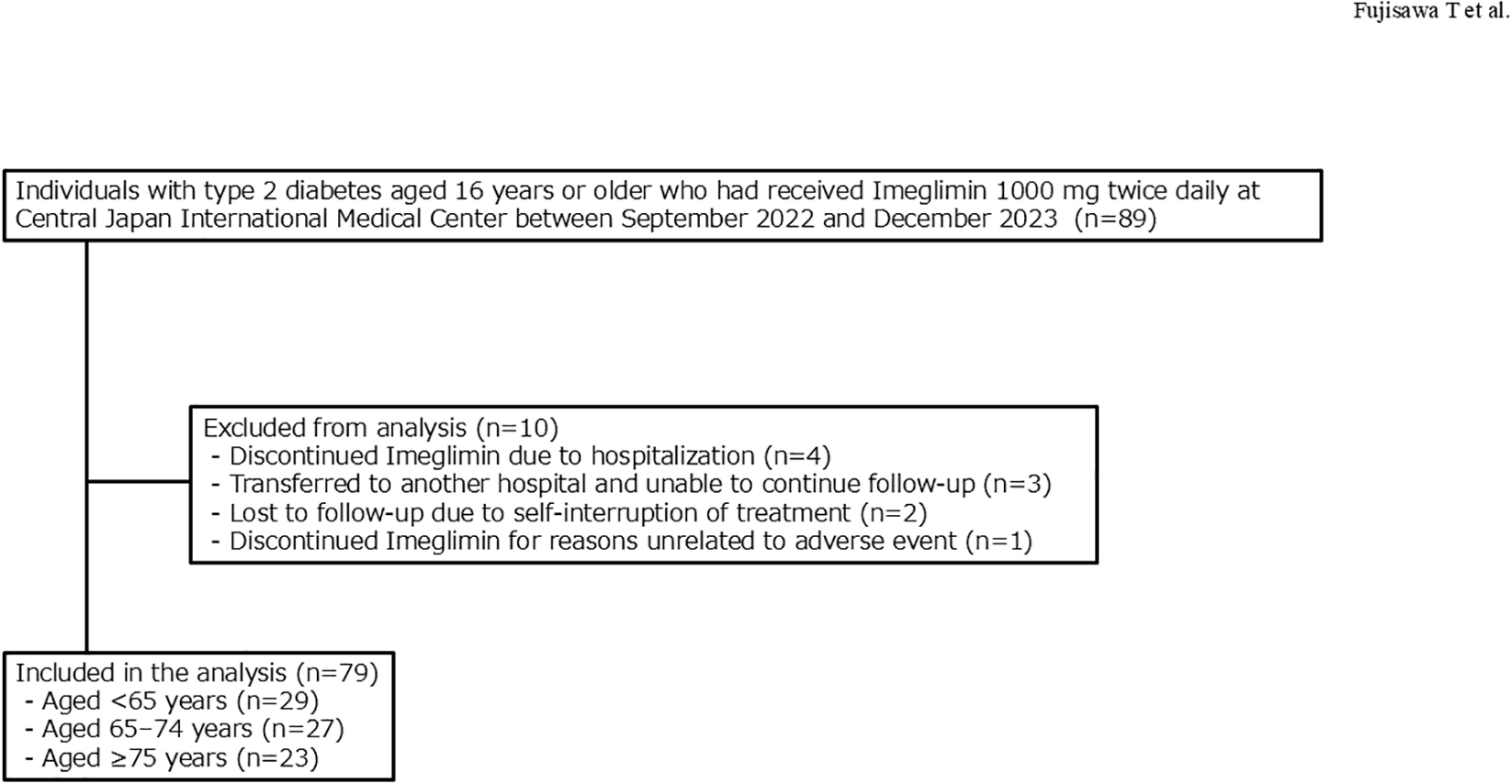
Flow diagram of enrollment and exclusion. Between September 2022 and December 2023, 89 individuals aged ≥16 years with type 2 diabetes who newly initiated imeglimin at 1,000 mg twice daily (2,000 mg/day) at the Central Japan International Medical Center were screened. Based on the exclusion criteria, 10 individuals were excluded: 4 discontinued treatments due to hospitalizations for conditions unrelated to imeglimin, 3 were lost to follow-up after transferring to other hospitals, 2 discontinued due to self-interruption, and 1 discontinued at personal request for reasons unrelated to adverse events. The final analysis included 79 subjects, comprising 29 subjects <65 years, 27 subjects aged 65–74 years, and 23 subjects aged ≥75 years.

Of the 89 individuals initially enrolled, 10 were excluded: 4 discontinued imeglimin due to hospitalizations unrelated to adverse events, 3 were lost to follow-up after transferring to other hospitals, 2 discontinued by self-interruption, and 1 discontinued at personal request (not related to adverse events). The final analysis included 79 individuals (**Figure 1**), whose baseline characteristics are summarized in **Table 1**.

**Table 1:** Baseline characteristics of the study population.

Baseline characteristics of the study population	All cases (n=79)	<65 years (n=29)	65–74 years (n=27)	>75 years (n=23)	p-value
Age (years)	66.0 ± 12.3	52.4 ± 8.5	69.7 ± 13.6	78.8 ± 3.1	<0.001
%Male (%)	65.8	62.1	77.8	52.2	0.162
BMI (kg/m^2^)*	25.2 ± 4.2	27.5 ± 4.6	23.8 ± 3.7	23.7 ± 2.6	0.001
Duration of Diabetes (years)*	11.7 ± 10.1	7.6 ± 5.3	17.6 ± 13.6	14.3 ± 10.0	0.072
No. of anti-diabetes medications	2.5 ± 1.3	2.2 ± 1.4	2.5 ± 1.2	2.9 ± 1.4	0.233
HbA1c (%)	8.0 ± 1.2	8.1 ± 1.4	8.0 ± 1.0	8.0 ± 1.0	0.900
eGFR (mL/min/1.73m^2^)	71.3 ± 17.9	77.5 ± 20.2	67.9 ± 13.9	67.6 ± 16.7	0.139
LDL-cholesterol (mg/dL)*	108.4 ± 27.1	117.6 ± 28.2	104.3 ± 23.9	102.7 ± 26.6	0.140
Triglyceride (mg/dL)*	172.7 ± 106.6	183.3 ± 122.1	168.1 ± 113.0	165.8 ± 71.8	0.915
ALT (IU/L)*	27.0 ± 17.6	31.8 ± 23.1	24.2 ± 13.0	24.0 ± 11.1	0.850
AST (IU/L)*	24.7 ± 11.5	25.4 ± 13.8	24.6 ± 11.0	23.9 ± 8.2	0.638
Previous anti-diabetes medications, n (%)					
None	6 (7.6)	3 (10.3)	1 (3.7)	2 (8.7)	0.666
SU	14 (17.7)	2 (6.9)	7 (25.9)	5 (21.7)	0.136
Glinide	19 (24.1)	6 (20.7)	3 (11.1)	10 (43.5)	0.030
BG	49 (62.0)	18 (62.1)	15 (55.6)	16 (69.6)	0.620
TZD	2 (2.5)	1 (3.4)	0 (0)	1 (4.3)	0.746
αGI	9 (11.4)	3 (10.3)	2 (7.4)	4 (17.4)	0.611
SGLT2i	32 (40.5)	12 (41.4)	10 (37.0)	10 (43.5)	0.919
DPP-4i	34 (43.0)	14 (48.3)	11 (40.7)	9 (39.1)	0.812
GLP-1RA	26 (32.9)	7 (24.1)	10 (37.0)	9 (39.1)	0.454
Insulin	25 (31.6)	6 (20.7)	12 (44.4)	7 (30.4)	0.155

*As some individuals included in the study lacked non-essential information for inclusion, the number of individuals analyzed for the following items is as follows: BMI (All, n=78; <65 years, n=29; 65–74 years, n=26; ≥75 years, n=23), Duration of diabetes (All, n=51; <65 years, n=25; 65–74 years, n=10; ≥75 years, n=15), LDL-cholesterol (All, n=69; <65 years, n=24; 65–74 years, n=25; ≥75 years, n=20), Triglyceride (All, n=69; <65 years, n=24; 65–74 years, n=25; ≥75 years, n=20), ALT (All, n=74; <65 years, n=28; 65–74 years, n=25; ≥75 years, n=21), AST (All, n=74; <65 years, n=28; 65–74 years, n=25; ≥75 years, n=21). Categorical variables and continuous variables are expressed as frequency and mean ± SD, respectively. For comparisons among the three groups— <65 years, 65–74 years and ≥75 years —the Kruskal-Wallis test was used for age, BMI, duration of diabetes, number of anti-diabetes medication, HbA1c, eGFR, LDL-C, TG, ALT, and AST. Fisher’s exact test was used for sex and type of anti-diabetes medication. αGI, α-glucosidase inhibitor; BMI, body mass index; HbA1c, glycated hemoglobin; AST, aspartate aminotransferase; ALT, alanine aminotransferase; BG, biguanide; DPP-4i, dipeptidyl peptidase-4 inhibitor; eGFR, estimated glomerular filtration rate; GLP-1RA, glucagon-like peptide-1 receptor agonist; LDL, low-density lipoprotein; SGLT2i, sodium glucose co-transporter 2 inhibitor; SU, sulfonylurea; TZD, thiazolidinedione.

### HbA1c changes and age-stratified outcomes

**Figure 2:**
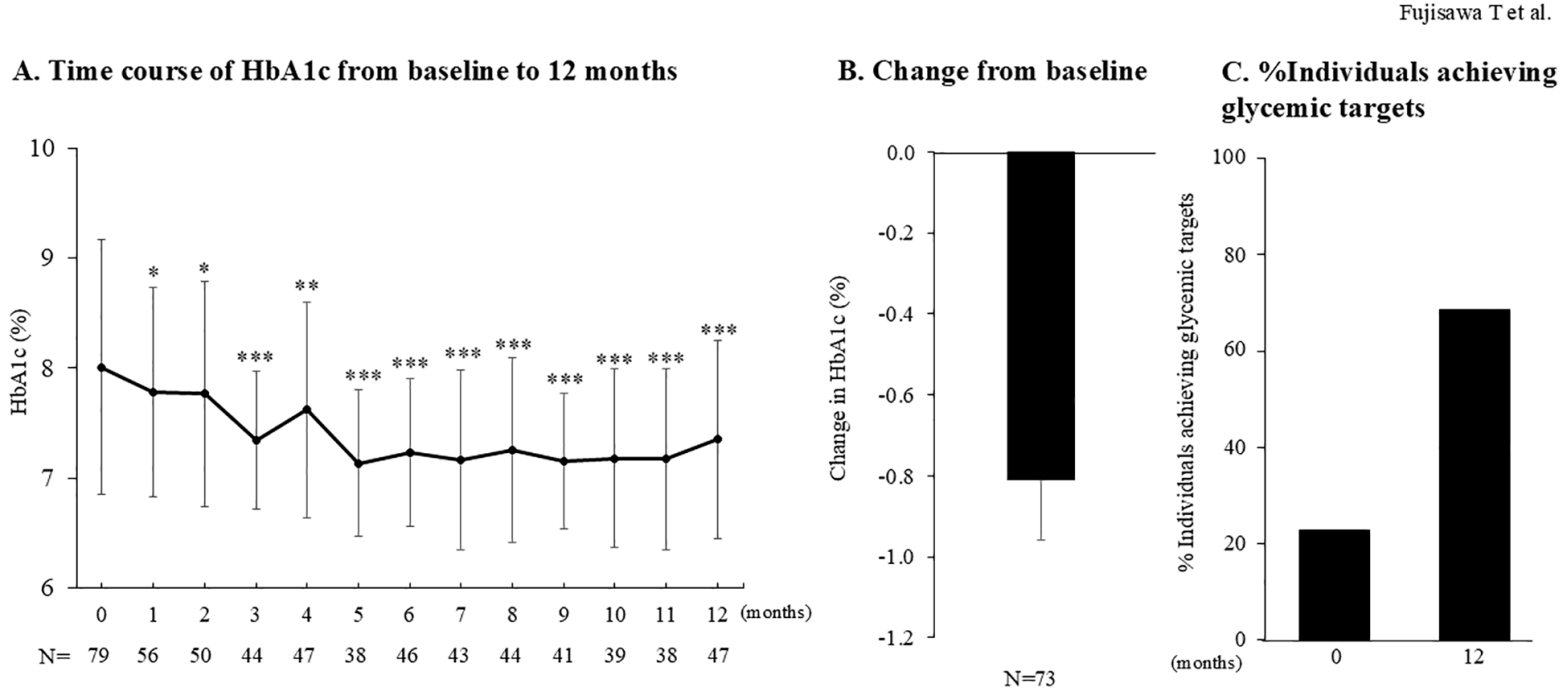
Changes in HbA1c during 12 months of imeglimin treatment. **(A)** Time course of HbA1c from baseline to 12 months. HbA1c trajectories are shown, with comparisons against baseline performed using the Wilcoxon signed-rank test. *p < 0.05 *vs*. baseline; **p<0.01 *vs*. baseline; ***p < 0.001 *vs*. baseline. **(B)** Change in HbA1c from baseline to 12 months. Data represents all subjects with available observations through week 52 (N = 73). **(C)** Proportion of subjects achieving glycemic targets at baseline (n= 9) and at 12 months (n=73). Glycemic targets were defined as HbA1c <7.0% for individuals <65 years, and for those ≥65 years, according to the joint recommendations of the Japanese Diabetes Society and the Japan Geriatrics Society.

The trajectory of HbA1c during the 12 months following imeglimin initiation is shown in **Figure 2**. HbA1c declined significantly as early as 1 month after initiation, with further improvement by 3 months. This effect was sustained throughout the 12-month period, resulting in a mean reduction of −0.8 ± 1.2% from baseline (**Figure 2**).

**Figure 3:**
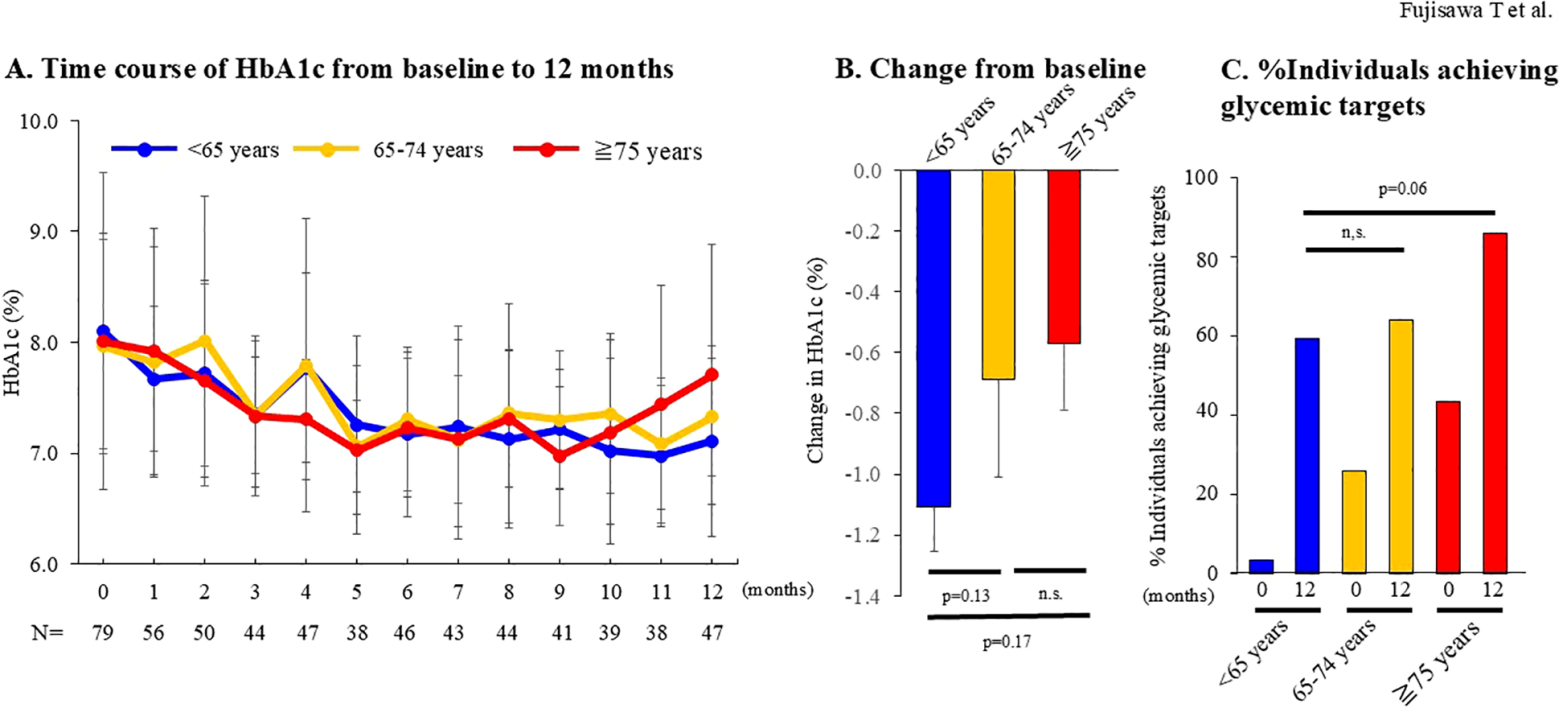
Comparison of effectiveness of imeglimin among individuals aged <65, 65–74, and ≥75 years. **(A)** Time course of HbA1c from baseline to 12 months in subjects aged <65 years (N = 29, blue line), 65–74 years (n=27, yellow line), and ≥75 years (n=23, red line). Comparisons of HbA1c trajectories among the three groups were performed using repeated-measures ANOVA. **(B)** Change in HbA1c from baseline to 12 months in subjects aged <65 years (n=27), 65–74 years (n=25), and ≥75 years (n=21). Between-group comparisons were conducted using the Mann-Whitney U test. **(C)** Proportion of subjects achieving glycemic targets at baseline and at 12 months in those aged <65 years (n=27), 65–74 years (n=25), and ≥75 years (n=21). Glycemic targets were defined as HbA1c <7.0% for individuals <65 years, and for those ≥65 years, according to the joint recommendations of the Japanese Diabetes Society and the Japan Geriatrics Society. Between-group comparisons of target achievement rates were performed using Fisher’s exact test.

At baseline, 29 individuals (36.7%) were <65 years, 27 (34.2%) were 65–74 years, and 23 (29.1%) were ≥75 years. Stratified analysis by age group showed that BMI was significantly higher in individuals <65 years, while concomitant use of glinides was more frequent in those ≥75 years; no other significant differences were observed (**Table 1**). The mean HbA1c change at 12 months was -1.1 ± 1.6% in those <65 years, -0.7 ± 1.1% in those 65–74 years, and -0.6 ± 0.7% in those ≥75 years. Although younger individuals demonstrated a numerically greater reduction, repeated-measures ANOVA revealed no significant differences among the three groups (p = 0.365; **Figures 3A, B**).

The proportion of participants achieving glycemic targets at 12 months (defined as HbA1c <7.0% for those <65 years, and age-specific targets per Japanese guidelines for those ≥65 years) was 59%, 64%, and 86% in the <65, 65-74, and ≥75 years groups, respectively. Although not statistically significant, a trend toward higher achievement rates was observed in the ≥75 years group compared with those <65 years (p = 0.06; **Figure 3C**).

### Effect of biguanide dose reduction

Among 49 individuals receiving biguanides at imeglimin initiation, 24 (49.0%) underwent dose reduction or discontinuation (reduction group), whereas 25 (51.0%) continued without dose reduction (non-reduction group). Baseline characteristics are summarized in **Supplementary Table S1**. The non-reduction group was significantly younger and had a higher BMI, but no other significant differences were observed.

**Figure 4:**
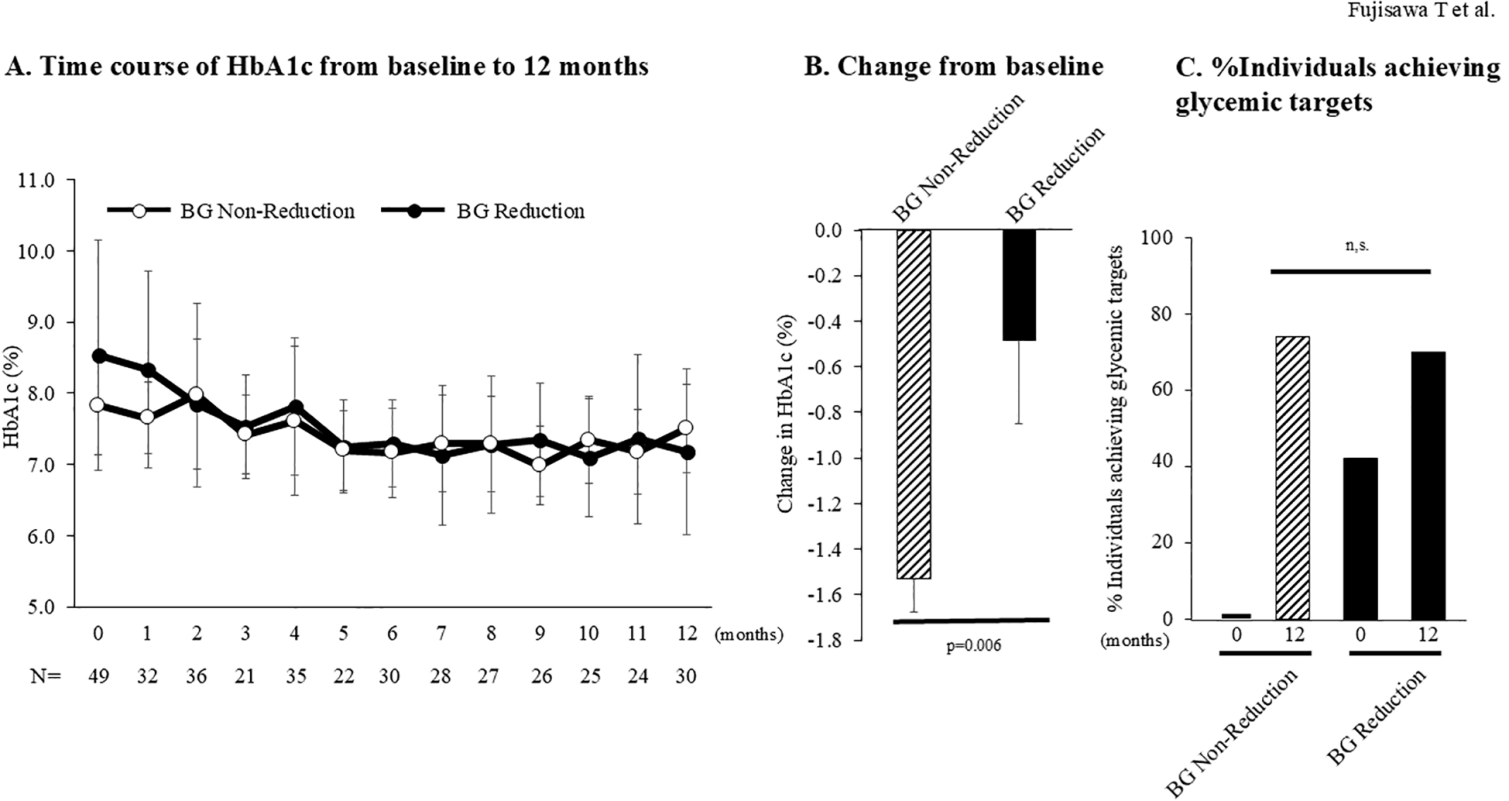
Comparison of effectiveness of imeglimin in individuals with and without biguanide (BG) dose reduction. **(A)** HbA1c trajectory from baseline to 12 months in the BG reduction/discontinuation group (n=24, dotted line) and BG non-reduction group (n=25, solid line). Comparisons of HbA1c trajectories among the two groups were performed using repeated-measures ANOVA. HbA1c trajectories between the two groups were compared using repeated-measures ANOVA. **(B)** Change in HbA1c from baseline to 12 months in all the BG non-reduction group (n=23), and the BG reduction group (n=23). Between-group comparisons were performed using the Mann–Whitney U test. **(C)** Proportion of subjects achieving glycemic targets at baseline and at 12 months in BG non-reduction group (n=23), and the BG reduction group (n=23). Glycemic targets were defined as HbA1c <7.0% for individuals <65 years, and for those ≥65 years, according to the joint recommendations of the Japanese Diabetes Society and the Japan Geriatrics Society. Between-group comparisons of target achievement rates were performed using Fisher’s exact test. BG, biguanide.

At 12 months, HbA1c decreased by -0.5 ± 0.7% in the reduction group and by -1.5 ± 1.7% in the non-reduction group. A significantly greater reduction was observed in the non-reduction group, confirmed by repeated-measures ANOVA (p=0.019; **Figures 4A–C**). The proportion of individuals achieving glycemic targets at 12 months was comparable between groups (70% *vs* 74%, p=1.000). However, while 42% of the reduction group achieved glycemic targets at baseline, none in the non-reduction group had done so, suggesting a greater relative improvement in the latter.

### Adverse events

**Figure 5:**
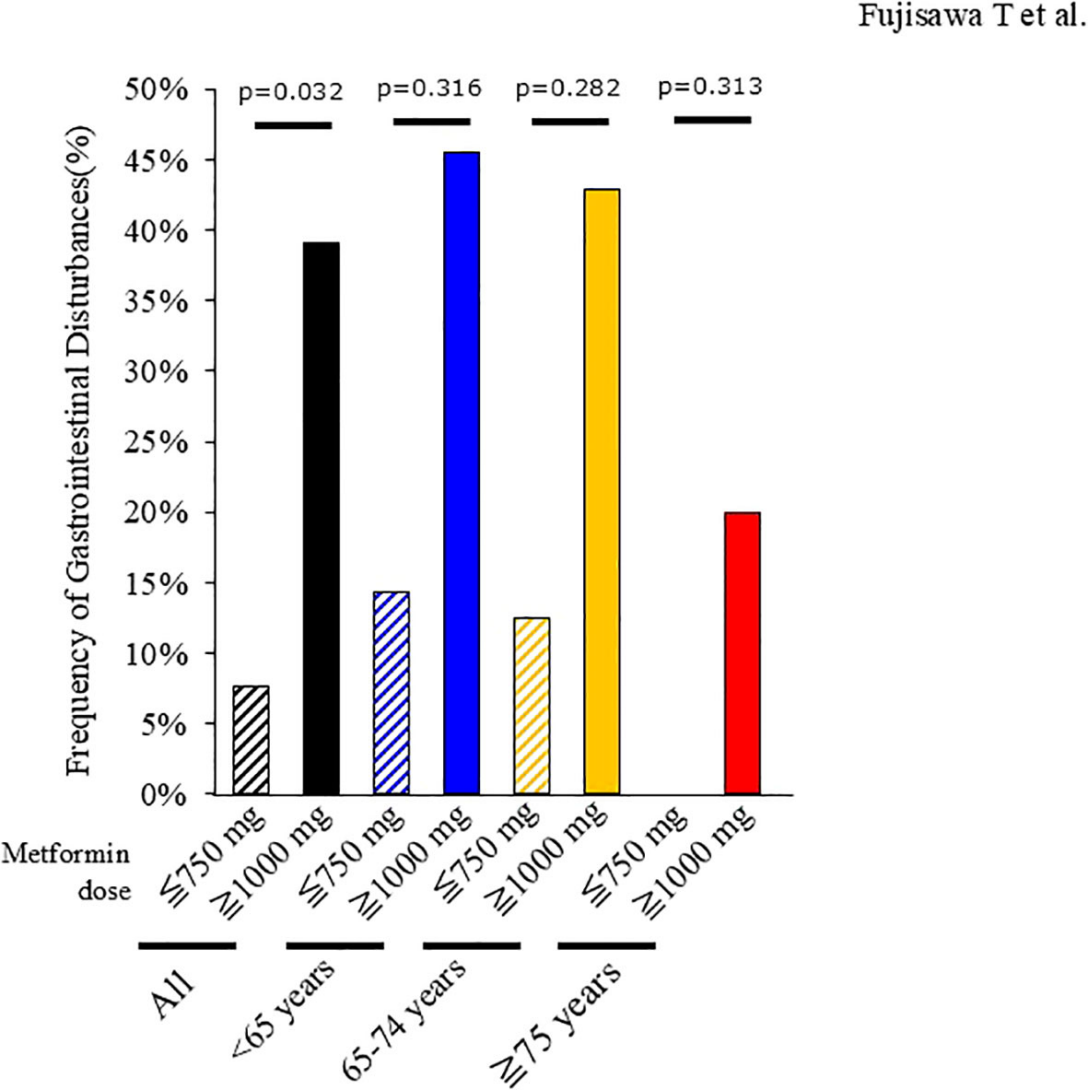
Relationship between gastrointestinal disturbances and biguanide (BG) dosage at imeglimin initiation. Among 49 subjects receiving concomitant BG at the time of imeglimin initiation, 26 received metformin ≤750 mg/day (<65 years: 7, 65–74 years: 8, ≥75 years: 11), and 23 received metformin ≥1,000 mg/day (<65 years: 11, 65–74 years: 7, ≥75 years: 5). The incidence of gastrointestinal adverse events was compared between groups using Fisher’s exact test. BG, biguanide.

Gastrointestinal (GI) disturbances, including nausea, vomiting, and constipation, occurred in 17 of 79 individuals (21.5%) (**Table 2**). Six patients (7.6%) discontinued imeglimin due to adverse events, including three cases related to GI symptoms. The incidence of GI disturbances by age group was 31.0% (9/29) in those <65 years, 22.2% (6/27) in those 65–74 years, and 8.7% (2/23) in those ≥75 years. Discontinuation due to adverse events occurred in 6.9%, 7.4%, and 8.7% of the respective groups. Neither the incidence of GI disturbances (p=0.139) nor discontinuation rates (p=1.000) differed significantly among age groups (**Table 3**). The frequency of GI disturbances was also comparable between the reduction and non-reduction groups (p=1.000; **Table 2**). However, when participants receiving biguanides were stratified by metformin dose (≤750 mg/day, n = 26 vs. ≥1,000 mg/day, n = 23), those receiving higher doses experienced significantly more GI disturbances (p=0.032; **Figure 5**). Similar trends were observed across all age groups, although statistical significance was not reached (<65 years, p=0.316; 65–74 years, p=0.282; ≥75 years, p=0.313; **Figure 5**).

**Table 2:** Adverse events in subjects with and without biguanide (BG) dose reduction.

Adverse events in subjects with and without biguanide (BG) dose reduction	All cases (n=79)	BG reduction (n=24)	BG non-reduction (n=25)	p-value
All AE (%)	21 (26.6)	7 (29.2)	6 (24.0)	0.754
Gastrointestinal disturbance (%)	17 (21.5)	5 (19.2)	6 (24.0)	1.000
Nausea (%)	6 (7.6)	1 (4.2)	3 (12.0)	0.609
Vomiting (%)	1 (1.3)	0 (0)	0 (0)	N/D
Diarrhea (%)	4 (5.1)	2 (8.3)	1 (4.0)	0.609
Constipation (%)	4 (5.1)	2 (8.3)	0 (0)	0.235
Reflux symptoms (%)	2 (2.5)	0 (0)	2 (8.0)	0.490
Hypoglycemia (%)				
Severe (%)	0 (0)	0 (0)	0 (0)	N/D
Non-severe (%)	2 (2.5)	1 (4.2)	0 (0)	0.490
Loss of hunger (%)	1 (1.3)	0 (0)	0 (0)	N/D
Nummular eczema (%)	1 (1.3)	1 (4.2)	0 (0)	0.490
Discontinuation due to AE (%)	6 (7.6)	1 (4.2)	2 (8.0)	1.000

Data are shown as absolute numbers and frequencies. Subjects were classified into the BG reduction group (N = 24) and BG non-reduction group (N = 25). Group comparisons were performed using Fisher’s exact test. BG, biguanide; AE, adverse event.

**Table 3:** Adverse events of imeglimin in older adults.

Adverse events of imeglimin in older adults	< 65 years (n=29)	65–74 years (n=27)	≧ 75 years (n=23)	p-value
All AE (%)	9 (31.0)	10 (37.0)	3 (13.0)	0.157
Gastrointestinal disturbance (%)	9 (31.0)	6 (22.2)	2 (8.7)	0.139
Nausea (%)	2 (6.9)	3 (11.1)	1 (4.3)	0.764
Vomiting (%)	1 (3.4)	0 (0)	0 (0)	1.000
Diarrhea (%)	2 (6.9)	1 (3.7)	1 (4.3)	1.000
Constipation (%)	2 (6.9)	2 (7.4)	0 (0)	0.545
Reflux symptoms (%)	2 (6.9)	0 (0)	0 (0)	0.328
Hypoglycemia (%)				
Severe (%)	0 (0)	0 (0)	0 (0)	N/D
Non-severe (%)	0 (0)	2 (7.4)	0 (0)	0.196
Loss of hunger (%)	0 (0)	0 (0)	1 (4.3)	0.291
Nummular eczema (%)	0 (0)	1 (3.7)	0 (0)	0.633
Discontinuation due to AE (%)	2 (6.9)	2 (7.4)	2(8.7)	1.000

Data are shown as absolute numbers and frequencies. Subjects were stratified into three groups: <65 years (n=29), 65–74 years (n=27), and ≥75 years (n=23). Group comparisons were performed using Fisher’s exact test. AE, adverse event.

Hypoglycemia occurred in 2 of 79 individuals (2.5%), none of which were severe. One case, a 71-year-old man receiving insulin, metformin, and a GLP-1 receptor agonist while on steroid replacement therapy for adrenal insufficiency, required discontinuation due to early-morning hypoglycemia.

### Other clinical parameters

In the 73 individuals who continued imeglimin (excluding 6 who discontinued due to adverse events), significant improvements were observed after 12 months in body weight (p=0.013), HbA1c (p<0.001), ALT (p<0.001), AST (p<0.001), γ-GTP (p<0.001), and triglycerides (p=0.001) (**Table 4**). Age-stratified analysis showed a significant reduction in LDL-C among individuals <65 years (p=0.046; **Supplementary Figure S1**). No other significant age-related differences were observed in changes in body weight, HbA1c, LDL-C, TG, ALT, AST, γ-GTP, or eGFR over 12 months.

**Table 4:** Changes in clinical parameters from baseline to 12 months after imeglimin initiation.

Changes in clinical parameters from baseline to 12 months after imeglimin initiation	n	Baseline	12 months	p value
Body weight (kg)	73	66.3 ± 14.2	65.7 ± 14.2	0.013
Systolic blood pressure (mmHg)	69	135.9 ± 16.6	135.3 ± 18.3	0.812
Diastolic blood pressure (mmHg)	69	72.5 ± 12.5	72.1 ± 11.8	0.673
Pulse (bpm)	51	81.6 ± 13.5	82.2 ± 13.3	0.819
HbA1c (%)	73	8.0 ± 1.2	7.2 ± 0.8	<0.001
LDL-cholesterol (mg/dL)	60	109.8 ± 26.1	104.2 ± 27.5	0.195
HDL-cholesterol (mg/dL)	59	57.4 ± 13.9	56.6 ± 11.6	0.423
Triglycerides (mg/dL)	60	178.8 ± 111.3	144.9 ± 74.0	0.001
ALT (IU/L)	66	27.2 ± 18.5	21.6 ± 12.2	<0.001
AST (IU/L)	66	25.1 ± 12.1	21.3 ± 8.1	<0.001
Fibrosis-4 index	45	1.52 ± 0.54	1.44 ± 0.53	0.06
γGTP (IU/L)	61	45.7 ± 49.7	34.8 ± 39.5	<0.001
Uric acid (mg/dL)	39	5.2 ± 1.4	5.1 ± 1.2	0.718
Creatinine (mg/dL)	73	0.80 ± 0.21	0.80 ± 0.22	0.861
eGFR (mL/min/1.73m^2^)	73	72.1 ± 18.3	71.7 ± 18.3	0.769

Data are presented as mean ± SD and absolute numbers. Comparisons between baseline and 12-month values were performed using the Wilcoxon signed-rank test. AST, aspartate aminotransferase; ALT, alanine aminotransferase; eGFR, estimated glomerular filtration rate; HDL, high-density lipoprotein; LDL, low-density lipoprotein.

## Discussion

In this retrospective study, we evaluated the real-world efficacy and safety of imeglimin administered at 1,000 mg twice daily (2,000 mg/day) for 12 months. The strengths and novelty of this study lie in demonstrating that Imeglimin showed sustained efficacy and favorable tolerability over 12 months in patients with type 2 diabetes regardless of age, and that the incidence of gastrointestinal adverse events was higher when it was combined with metformin at doses of ≥1,000 mg/day. Our findings demonstrated a significant reduction in HbA1c along with favorable tolerability. Imeglimin has been shown in the Japanese Phase 3 clinical trials (TIMES 1-3) to significantly reduce HbA1c both as monotherapy and in combination with other agents (4–6). The present study confirmed these results in routine clinical practice. Furthermore, since only six participants (7.6%) received imeglimin monotherapy, our findings primarily reflect combination therapy, including with insulin, supporting its additive glycemic effect observed in previous studies such as the FAMILIAR trial interim analysis and the MEGMI study (10, 11). Notably, the glucose-lowering effect of imeglimin appeared somewhat gradual, becoming more pronounced after 3 months of treatment and sustained through 12 months. This pattern is consistent with the findings of Osonoi et al. (12) and with established evidence on the divergence between HbA1c and glycated albumin trajectories (13), highlighting that imeglimin requires time to exert its full effect.

More than 60% of the individuals in this study were ≥65 years of age, reflecting the increasing proportion of older individuals with type 2 diabetes in Japan. While subgroup analyses from the TIMES trials reported significant HbA1c reductions in older adults, real-world data remain scarce. In our age-stratified analysis (<65, 65–74, and ≥75 years), HbA1c reductions, glycemic trajectories, and target achievement rates did not differ significantly among groups. However, individuals aged ≥75 years demonstrated a trend toward higher glycemic target achievement compared with those <65 years. Importantly, the frequency of adverse events and treatment discontinuation did not differ among age groups, and the incidence of gastrointestinal events was numerically lower in the ≥75 years group. These findings suggest that the effectiveness and safety of imeglimin in older adults, including those aged ≥75 years, is comparable to that observed in younger populations, underscoring its potential as a treatment option for the growing population of older individuals with type 2 diabetes. Moreover, Imeglimin and metformin share a similar mechanism involving inhibition of mitochondrial complex I, which suppresses hepatic gluconeogenesis. However, imeglimin acts as a competitive inhibitor, thereby reducing the risk of lactic acidosis compared with metformin (14). Consistent with this mechanism, no cases of lactic acidosis were observed in our study, even among elderly individuals aged ≥65 years receiving combination therapy with metformin. In Addition, our study also provides clinically relevant evidence in very old adults (≥75 years), who are often underrepresented in clinical trials. Indeed, 23 participants (29.1%) were ≥75 years, supporting imeglimin utility even in this vulnerable subgroup. These results are consistent with the FAMILIAR trial interim analysis by Kaku et al. (10), which demonstrated comparable efficacy and safety of imeglimin in individuals aged <65 and ≥65 years in a prospective multicenter design. Nevertheless, large-scale and long-term prospective studies are warranted to clarify the long-term effects of imeglimin, particularly in the very elderly.

Another important finding relates to the concomitant use of biguanides. Biguanides are frequently prescribed in current diabetes treatment, and the effectiveness of concomitant use of biguanides with Imeglimin has been reported in the MEGMI study by Takahashi et al. (11). The TIMES 2 trial reported a higher incidence of Gl adverse events when imeglimin was combined with biguanides (5). Although the underlying mechanism remains unclear, both agents are known to inhibit mitochondrial Complex I and share structural similarities, which may contribute to gastrointestinal intolerance (2). Animal studies have further shown that imeglimin alters the gut microbiota in a manner similar to metformin (15), potentially exacerbating gastrointestinal disturbances. In our stratified analysis, HbA1c reduction was significantly greater in individuals who continued biguanides without dose reduction compared with those who underwent dose reduction or discontinuation. While glycemic target achievement rates were similar between groups, baseline attainment was higher in the reduction group, suggesting a more pronounced relative improvement in the non-reduction group. Regarding safety, gastrointestinal adverse events did not differ between groups. However, further stratification by metformin dose revealed a significantly higher incidence of gastrointestinal events among those receiving ≥1,000 mg/day. These findings suggest that imeglimin may be safely combined with biguanides at <1,000 mg/day, whereas higher doses may increase gastrointestinal risk.

Beyond glycemic control, additional benefits of diabetes therapies are increasingly emphasized. Imeglimin, as a first-in-class agent, has been investigated for its potential effects beyond glucose lowering. Among comorbidities, metabolic dysfunction-associated steatotic liver disease (MASLD) is particularly important, and recent treatment algorithms from both the American Diabetes Association/he European Association for the Study of Diabetes and the Japan Diabetes Society recommend considering MASLD when selecting antidiabetic therapies (16, 17). MASLD is associated not only with hepatic outcomes such as cirrhosis and hepatocellular carcinoma but also with atherosclerotic cardiovascular disease, heart failure, and certain extrahepatic malignancies (18). Its prevalence in type 2 diabetes exceeds 50%, more than double that observed in non-diabetic individuals (19), highlighting the need for effective therapies. Imeglimin has been identified as a potential hepatoprotective agent. *In vitro* studies in rat hepatocytes demonstrated dose-dependent suppression of gluconeogenesis (14), while *in vivo* studies in non-diabetic metabolic dysfunction-associated steatohepatitis (MASH) mouse models showed that imeglimin attenuates disease progression independent of its glucose-lowering effect by improving mitochondrial function, enhancing fatty acid oxidation, and reducing reactive oxygen species (20). Additionally, Kikuchi et al. reported that imeglimin improved hepatic steatosis in high-fat, high-sucrose diet-fed mice by enhancing glucagon secretion and thereby suppressing lipogenesis and promoting fatty acid oxidation (21). Clinical evidence, though limited, includes case reports in individuals with type 2 diabetes and MAFLD demonstrating reductions in liver fat content after 6 months of imeglimin therapy (22). The MEGMI study reported that treatment with Imeglimin significantly improved liver enzyme elevations and the fatty-liver index, and there was a significant correlation between the extent of HbA1c reduction and improvement in fatty-liver disease indicators (11). In our study, although direct measures of hepatic steatosis were not performed, significant reductions in liver enzymes (i.e., ALT, AST and γ-GTP) were observed after 12 months, suggesting a potential hepatoprotective effect. These findings warrant further large-scale and long-term studies to evaluate imeglimin’s role in MASLD. In this study, a significant reduction in HbA1c was observed even in non-older individuals (<65 years) who had a significantly higher BMI. Fukunaga et al. have demonstrated that imeglimin treatment in individuals with type 2 diabetes complicated by obesity and fatty liver significantly improved glycemic control as well as liver-related outcomes (23). The present finding that imeglimin was also effective in non-older individuals with obesity and type 2 diabetes suggests that imeglimin may be a useful therapeutic option for diabetes in populations where obesity is a predominant feature. Further validation in larger-scale studies is warranted. Beyond its effects on hepatic parameters, imeglimin may also confer systemic metabolic benefits through mitochondrial and inflammatory pathways. Satheesan et al. reported that imeglimin-based therapies reduced circulating cell-free mitochondrial DNA and proinflammatory cytokines in individuals with type 2 diabetes, suggesting attenuation of mitochondrial stress and low-grade inflammation (24). Although our study did not assess such biomarkers, the observed improvements in triglycerides, liver enzymes, and body weight may be consistent with these additional metabolic benefits beyond glycemic control.

### Limitations

This study has several limitations. First, it was a single-center retrospective observational study with a relatively small sample size, which may limit the generalizability of the findings. Second, the study population was almost entirely Japanese, which restricts external validity to other ethnic groups. Although our study was a single-center retrospective analysis, similar limitations in sample size and ethnicity have been reported in multicenter prospective trials of imeglimin (10, 11). Third, as the analysis was based on routine clinical records, concomitant medication changes may have confounded the results, and thus the observed effects cannot be attributed solely to imeglimin. Nevertheless, given that imeglimin was only approved in September 2021, real-world evidence regarding its long-term effectiveness and safety remains limited. Our findings therefore provide valuable insights into its clinical use and outcomes in everyday practice.

## Conclusion

In conclusion, imeglimin demonstrated sustained effectiveness and good tolerability over 12 months in both older and younger individuals with type 2 diabetes, with no significant differences in glycemic effect or adverse event incidence between age groups. Importantly, imeglimin appeared effective and safe even in individuals aged ≥75 years. While gastrointestinal adverse events did not differ by biguanide dose reduction status, metformin doses ≥1,000 mg/day were associated with a higher frequency of such events. Future large-scale prospective studies are needed to further clarify the effectiveness and safety of imeglimin in older adults, particularly the very elderly, and to determine optimal strategies for co-administration with biguanides. Overall, imeglimin represents a promising first-in-class oral antidiabetic agent with potential not only for glucose lowering but also for broader metabolic benefits in routine clinical practice.

## Data Availability

The raw data supporting the conclusions of this article will be made available by the authors, without undue reservation.

## Supplementary material

Supplementary Figure 1 Changes in clinical parameters after imeglimin initiation: Comparison between older and non-older individuals.
