# Supplementary figures and images for: Real-world effectiveness and safety of imeglimin: a single-center retrospective cohort study in Japan
Source: Front Clin Diabetes Healthc. 2025 Dec 16;6:1694522. doi: 10.3389/fcdhc.2025.1694522 (PMC12747980; doi:10.3389/fcdhc.2025.1694522)

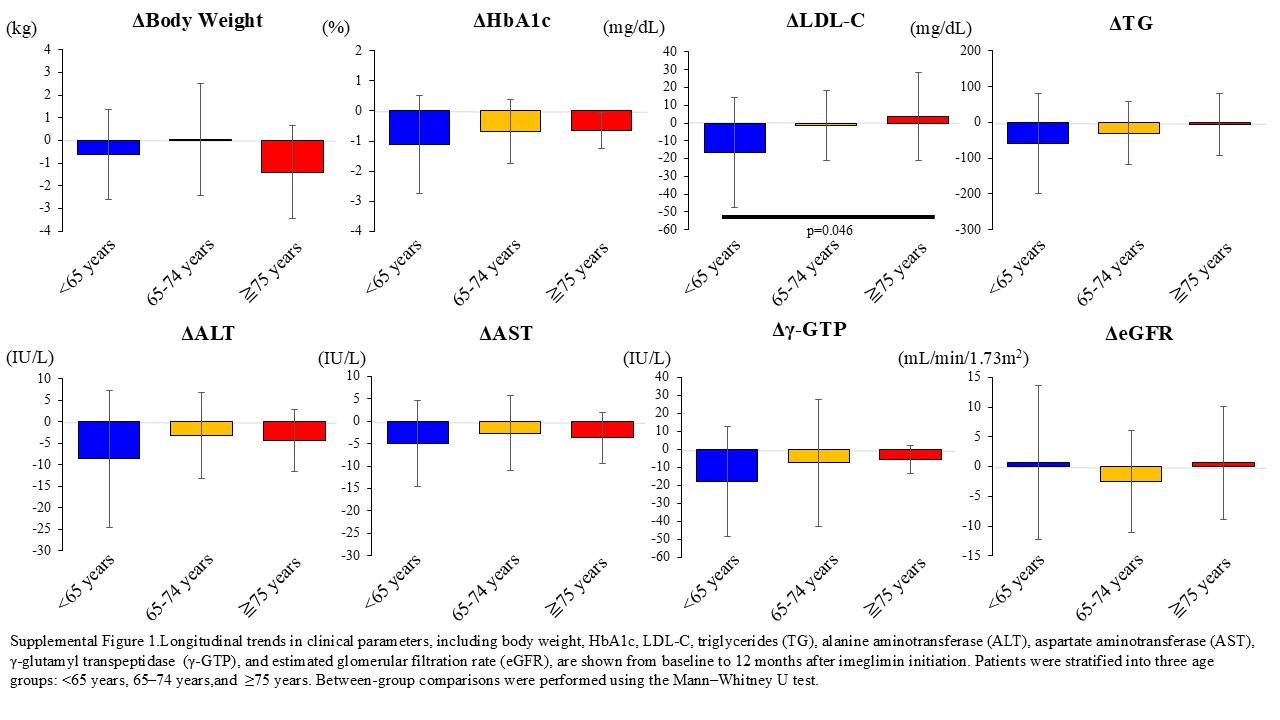

Supplement: Supplementary Figure 1 — Changes in clinical parameters after imeglimin initiation: Comparison between older and non-older individuals. [file Image1.jpeg]
