# Supplementary material for: Real-world effectiveness and safety of imeglimin: a single-center retrospective cohort study in Japan
Source: Front Clin Diabetes Healthc. 2025 Dec 16;6:1694522. doi: 10.3389/fcdhc.2025.1694522 (PMC12747980; doi:10.3389/fcdhc.2025.1694522)
Supplement: Supplementary file 2 [file Table1.docx]

**Supplementary Table 1. Baseline characteristics of individuals with and without biguanide reduction.**

|  | All cases  (N=49) | BG Non-Reduction  (N=25) | BG Reduction  (N=24) | p-value |
| --- | --- | --- | --- | --- |
| Baseline BG dosage (mg) | 1045.9±393.4 | 1050.0±441.6 | 1041.7±335.9 | 0.942 |
| BG dosage initiating Imeglimin (mg) | 729.6±536.5 | 1050.0±441.6 | 395.8±407.7 | <0.001 |
| Age (years) | 65.5±13.7 | 60.5±13.3 | 70.7±12.2 | 0.009 |
| %Male (%) | 63.3 | 68.0 | 58.3 | 0.561 |
| BMI (kg/m^2^) | 25.1±4.2 | 26.4±4.2 | 23.8±3.7 | 0.028 |
| Duration of Diabetes  (years)* | 13.2±10.7 | 11.5±9.2 | 15.8±12.2 | 0.3 |
| No. of anti-diabetes  medications | 3.0±1.1 | 3.2±1.2 | 2.8±1.1 | 0.21 |
| HbA1c (％) | 8.2±1.3 | 8.5±1.6 | 7.8±0.7 | 0.062 |
| eGFR (mL/min/1.73m^2^) | 72.1±18.1 | 73.6±18.0 | 70.5±17.9 | 0.552 |
| LDL-cholesterol (mg/dL)* | 107.4±29.5 | 112.2±33.2 | 103.9±24.8 | 0.441 |
| Triglyceride (mg/dL)* | 174.8±116.5 | 201.8±153.6 | 149.1±51.6 | 0.156 |
| ALT (IU/L)* | 28.1±18.7 | 31.2±17.2 | 25.0±19.7 | 0.267 |
| AST (IU/L)* | 24.9±11.9 | 25.8±13.9 | 24.0±9.4 | 0.621 |

*As some individuals included in the study lacked non-essential information for inclusion, the number of individuals analyzed for the following items is as follows: Duration of diabetes (All, 31; BG Non-Reduction, 19; BG Reduction, 12) , LDL-cholesterol (All, 41; BG Non-Reduction, 20; BG Reduction, 21), Triglyceride (All, 41; BG Non-Reduction, 20; BG Reduction, 21), ALT (All, 46; BG Non-Reduction, 23; BG Reduction, 23), AST (All, 46; BG Non-Reduction, 23; BG Reduction, 23). Categorical variables and continuous variables are expressed as frequency and mean ± SD, respectively. For comparisons among the groups— BG reduction group and BG non-reduction group —the Independent t-test was used for age, BMI, duration of diabetes, number of anti-diabetes medication, HbA1c, eGFR, LDL-C, TG, ALT, and AST. Fisher’s exact test was used for sex. BMI, body mass index; HbA1c, glycated hemoglobin; AST, aspartate aminotransferase; ALT, alanine aminotransferase; eGFR, estimated glomerular filtration rate; LDL, low-density lipoprotein.
